# The effectiveness of generalist (GIT-PD) versus specialist treatment (MBT/ST) for severe personality disorders (Personality Disorders Access to Effective Treatment, P-DAET): study protocol of a pragmatic randomised controlled non-inferiority multicentre trial

**DOI:** 10.1186/s12888-025-07550-4

**Published:** 2025-11-20

**Authors:** Christel Bomhof, Jan Philipp Löffler, Suzanne Brugman, Paul Lodder, Arne van den End, Maarten Kornelis van Dijk, Maarten van Westen, Loek van Dam, Ilse Wielaard, Wendy Mensink, Lieke Muskens, Bregje de Moor, Nathan Bachrach, Henricus L. Van, Joost Hutsebaut

**Affiliations:** 1 Viersprong Institute for Studies on Personality Disorders, Halsteren, The Netherlands; 2 Tilburg University, Tilburg, The Netherlands; 3 Arkin NPI, P.O. Box 7031, Amsterdam, JA 1007 The Netherlands; 4 GGZ Oost Brabant, Helmond, The Netherlands; 5 Arkin NPI, Amersfoort, The Netherlands; 6 Dimence Group, Deventer, The Netherlands; 7 Altrecht, Utrecht, The Netherlands; 8 Dimence Group, Almelo, The Netherlands; 9 GGZ Oost Brabant, Oss, The Netherlands; 10 GGZ Oost Brabant, Boxmeer, The Netherlands

**Keywords:** Severe personality disorders, Generalist psychotherapy, Specialist psychotherapy, Randomised controlled trial, GIT-PD, ST, MBT, Non-inferiority

## Abstract

**Background:**

Personality disorders (PDs) are highly prevalent, impairing, and societally burdening conditions which can be treated effectively. Specialist psychotherapies are the first-choice treatment, but accessibility is hampered by implementation and sustainability issues. Manualised generalist treatments were designed as active control conditions to specialist psychotherapies in several effectiveness trials; their comparable performance led to them being regarded as more accessible alternatives to specialist psychotherapies. The Guideline-Informed Treatment for PDs (GIT-PD) is such a generalist treatment, which has shown promise and is broadly disseminated in the Netherlands and Belgium. However, there is an urgent need for controlled head-to-head comparisons of generalist and specialist treatments for PDs. Therefore, this study poses three main questions: 1) Is generalist treatment non-inferior compared to specialist treatment for severe PDs? 2) Is generalist treatment cost-effective compared to specialist treatment? 3) Which combination of patient characteristics predicts differential treatment outcomes between generalist and specialist treatments?

**Methods:**

P-DAET is a pragmatic multicentre randomised controlled non-inferiority trial. In total, 358 adult treatment-seeking participants with severe PDs will be included in five Dutch mental healthcare institutions. Participants are equally randomised to manualised generalist (GIT-PD) or specialist treatment (Mentalisation-Based Treatment/Schema Therapy). The primary outcome is improvement in personality functioning, assessed through assessor-blinded clinical interviews and supplemented with self-report questionnaires. Symptom severity and psychosocial functioning are important secondary outcomes. Participants complete assessments at 7 time-points: baseline (T0), 3 (T1), 6 (T2), 12 (T3), 18 (T4), 24 (T5), and 30 months (T6) after the start of treatment. The primary endpoint is at 18 months. An economic evaluation will be executed alongside the RCT. Differential treatment response based on candidate predictors at the patient level will be explored using a personalised advantage index (PAI).

**Discussion:**

P-DAET will determine the (cost-)effectiveness of generalist versus specialist treatment for severe PDs. This study will provide novel insights into the applicability of these treatments and their differential treatment response, enabling effective and personalised allocation of limited specialist treatment resources, while contributing to more accessible and good-enough care for individuals with severe PDs.

**Trial registration:**

ClinicalTrials.gov: NCT06789380. Retrospectively registered on February 5, 2025.

**Supplementary information:**

The online version contains supplementary material available at 10.1186/s12888-025-07550-4.

**Table:** 

Administrative information	
Title {1a}	The effectiveness of generalist (GIT-PD) versus specialist treatment (MBT/ST) for severe personality disorders (Personality Disorders Access to Effective Treatment, P-DAET): study protocol of a pragmatic randomised controlled non-inferiority multicentre trial.
Protocol version {2}	Issue date: 27-Mar-2025 Protocol amendment number: 02 Authors: CAB, SB **Revision chronology:** P-DAET Protocol 00, 2024-May-23 Original P-DAET Protocol 01, 2024-Aug-05 Amendment 01.: Primary reason for amendment: changes in sections 3, 8.1.2, 10.2, and 12.6 regarding additional time points, changes in secondary outcomes, and an increase in monetary compensation. Additional changes (these changes in and of themselves would not justify a protocol amendment): supplying the correct version of the C-SSRS. P-DAET Protocol 02, 2025-Mar-27 Amendment 02.: Primary reason for amendment: changes in sections 2, 3, 8.1.1, 8.1.2, 8.2, 8.3, 10.1, 10.2, and 11.2 regarding statistical analysis of primary and secondary outcomes, permission to collect informed consent digitally, and specification of the randomisation procedure. Additional changes (these changes in and of themselves would not justify a protocol amendment): changes on the title page and in section 12.1 regarding changes in personnel at Altrecht, and provided the name of the clinical data management platform used.
Names, affiliations, and roles of protocol contributors {3a}	C. A. Bomhof: Viersprong Institute for Studies on Personality Disorders, Halsteren, The Netherlands; Tilburg University, Tilburg, The Netherlands J. P. Löffler: Arkin NPI, Amsterdam, The Netherlands; Tilburg University, Tilburg, The Netherlands S. Brugman: GGZ Oost Brabant, Helmond, The Netherlands P. Lodder: Tilburg University, Tilburg, The Netherlands A. A. van den End : Arkin NPI, Amersfoort, The Netherlands M. K. van Dijk: Dimence Group, Deventer, The Netherlands M. van Westen: Altrecht, Utrecht, The Netherlands L. J. H. van Dam: Arkin NPI, Amsterdam, The Netherlands I. Wielaard: Dimence Group, Almelo, The Netherlands W. Mensink: Altrecht, Utrecht, The Netherlands L. A. M. Muskens: GGZ Oost Brabant, Oss, The Netherlands B.M. de Moor: GGZ Oost Brabant, Boxmeer, The Netherlands N. Bachrach: GGZ Oost Brabant, Helmond, The Netherlands; Tilburg University, Tilburg, The Netherlands H. L. Van: Arkin NPI, Amsterdam, The Netherlands J. Hutsebaut: Viersprong Institute for Studies on Personality Disorders, Halsteren, The Netherlands; Tilburg University, Tilburg, The Netherlands
Name and contact information for the trial sponsor {3b}	The principal investigator (PI) and sponsor of the trial is Henricus L. Van (rien.van@arkin.nl; Medical Director at Arkin NPI; P.O. Box 7031, 1007 JA Amsterdam, the Netherlands).
Role of sponsor and funders {3c}	The PI and study sponsor, H.L. Van, was actively involved in the design and management of the study and co-supervised the writing of the report. The study funder was not actively involved in the design and execution of the study, nor was the funder involved in the writing of the report.
Composition of the coordinating centre and trial steering committee {3d}	A trial steering committee (TSC) consists of the principal investigator, a senior researcher per participating institution, two junior researchers, and site coordinators per participating site. The TSC has monthly meetings to discuss trial conduct, participant recruitment and inclusion, (serious) adverse events (SAEs), and publication of trial results. Furthermore, part of the senior researchers simultaneously act as (PhD) supervisor for the junior researchers, who are responsible for the day-to-day execution of the trial and oversee research assistants. This smaller core team meets on a weekly basis. Key roles external to the TSC and the executive core team consist of the data manager, advisors on statistical analysis and methodology, and the independent experts whom participants can turn to for impartial advice.
Open science	
Trial registration {4}	ClinicalTrials.gov: NCT06789380 Retrospectively registered on February 5, 2025.
Protocol and statistical analysis plan {5}	The planned procedures and the statistical analysis plan are available in the current publication, as well as in part within the time-stamped protocol approved by the accredited medical ethical research commission (METC Brabant), accessible on Clinical Trials.gov. The dissemination of amendments is detailed under Protocol amendments {31}.
Dissemination policy {8}	In general, all results will be published in peer-reviewed national and international journals. In addition, all publications will be disseminated to the public through national and international presentations and lectures, as well as the websites and newsletters of participating institutes (Altrecht, Dimence Groep, GGZ Oost Brabant, Arkin NPI, and GGZ de Viersprong), and the dedicated P-DAET-trial website. Further dissemination of results to participants of the study will be discussed with patient boards. Depending on the interpretation of the results, this study may lead to modifications of the regular mental health practice at the participating institutes and beyond.

**Note**: The numbers in curly brackets in this protocol refer to SPIRIT 2025 checklist item numbers (Chan et al. 2025).

## Introduction

### Background and rationale {9a}

Personality disorders (PDs) represent a significant health concern worldwide, estimated to affect around one in ten individuals in the general population [1, 2], with substantially higher prevalence rates observed in psychiatric samples [3]. PDs are associated with severely impaired quality of life [4], co-occurring somatic diseases [5], diminished labour productivity, and increased healthcare costs. More specifically, societal costs of PDs are comparable to schizophrenia and significantly exceed those of depression, anxiety disorders, multiple sclerosis, and epilepsy [6–8]. Therefore, access to effective treatment programs is highly relevant to both individual patients and society as a whole.

International guidelines recommend specialist psychotherapy as the first-choice treatment for PDs [9], with substantial evidence supporting the use of Dialectical Behaviour Therapy (DBT), Mentalisation-Based Treatment (MBT), Schema Therapy (ST), and Transference Focused Psychotherapy (TFP) for borderline PD (BPD) [10–14]. These specialist psychotherapies have consistently and reliably outperformed treatment as usual (TAU), with no evidence for superiority of one over another [10–15]. Recent applications of these evidence-based therapies have also shown effectiveness in treating other personality disorders, such as antisocial, paranoid, narcissistic, and cluster-C PD [16–19].

However, the implementation and long-term sustainability of these specialist treatments in routine clinical practice face various challenges. Apart from overall constraints in healthcare financing and limited organisational funding [20], key challenges are high staff turnover [21] and a shortage of trained therapists for specialist programs [22]. In addition, these programs are often relatively costly due to intensive training requirements for clinicians, high treatment dosage, and extended duration. Following implementation, the long-term viability of specialist programs needs sustained organisational support, time allocation, and competent team leadership, all of which are not always available [23, 24]. Consequently, the availability of evidence-based specialist treatments remains inadequate to meet the demand for treatment of individuals with PDs [22].

A second generation of clinical trials may have fortuitously revealed a potentially more accessible alternative to specialist treatment. After the first generation of trials had demonstrated that borderline PD could, in fact, be treated effectively, subsequent trials failed to establish superiority of any one specialist treatment, despite adhering to fundamentally disparate models. To ensure methodological rigour and avoid withholding active treatment from trial participants, a new series of clinical trials was conducted with an enhanced manualised active control condition [25]. As a result, manualised generalist treatments for personality disorders, such as General Psychiatric Management (GPM), Structured Clinical Management (SCM), Good Clinical Care (GCC), and Supportive Therapy, were introduced in a handful of trials evaluating specialist treatments, like DBT, MBT, Cognitive Analytic Therapy (CAT), and TFP [26–29]. Unexpectedly, the specialist treatments did not outperform the generalist treatments. These findings were reinforced by meta-analyses indicating that manualised generalist treatments can be as effective as specialist therapies, with both demonstrating superior outcomes compared to TAU [10, 11]. Given the high prevalence of individuals with PDs seeking care and the limited availability of specialist psychotherapies, generalist treatments were therefore reconsidered as viable, accessible alternatives to specialist treatments [22, 25]. The issue is, however, not without controversy, as a recent network meta-analysis by Setkowski et al. [12] did not corroborate earlier meta-analyses.

More specifically, the generalist treatments incorporated the common non-specific factors found in specialist treatments, referring to a structured treatment framework, an active and supportive therapist stance, the utilisation of interventions aimed at explorative clarification, change encouragement, and empathic validation, as well as a focus on affect and the therapeutic relationship [30]. At the same time, generalist models address barriers associated with specialist treatments through their shorter duration, lower treatment intensity, reduced costs, reliance on less specialised therapists, and fewer demands for training and supervision [25]. These features could make generalist treatments far more scalable and cost-effective than specialist therapies [22]. Although some research has yielded tentatively promising results, few trials have been conducted directly comparing the two treatment conditions [10–12]. Furthermore, all previous trials were designed to examine superiority. As a result, to unambiguously conclude that generalist treatments represent a valuable alternative to specialist treatments for PD trials that demonstrate non-inferiority need to be conducted.

The Guideline-Informed Treatment for Personality Disorders (GIT-PD) offers an evidence-informed treatment framework, comparable to GPM and SCM. GIT-PD was developed in the Netherlands to enhance access to mental health care for individuals with PDs [31]. Hutsebaut et al. [31] describe GIT-PD as a principle-driven model designed to optimise common factors and effective treatment components, in a structured manner across four levels of implementation: The institution, the clinical pathway, the treatment team, and the individual therapist. Moreover, GIT-PD offers flexibility, allowing it to adapt to the specific clinical needs of individuals with PDs and the existing competencies of professionals. Compared to specialist treatments for PDs, GIT-PD is characterised by lower treatment intensity, shorter duration, reduced training and supervision requirements, and a less specialised therapist profile—all of which are expected to result in lower overall costs. A recent multicentre open cohort study based on routine outcome monitoring (ROM) demonstrated the feasibility of GIT-PD and suggested effect sizes comparable to those of most specialist treatments for PDs [32]. Lacking a control group and randomisation, the extent to which these findings are subject to bias and confounding remains unclear, warranting confirmation in a randomised controlled trial.

A limitation of the current evidence base surrounding treatments for PDs is its predominant reliance on average treatment effects. All the while, these average effects face substantial heterogeneity [10, 11, 13, 14, 33, 34], meaning that individual treatment responses vary greatly. As specialist treatments have failed to demonstrate superiority over generalist models at the group level, it has been suggested that differences between the two might be spurious, but specific subgroups of patients might benefit from specialist treatment [11, 25]. Therefore, personalised treatment selection is seen as a potentially favourable approach to optimise treatment response and resource allocation [33, 34] in which patient characteristics inform the selection of the most appropriate treatment [35, 36]. To date, research has sought to discern characteristics which can predict treatment response and has tentatively suggested some (for instance, psychiatric symptom severity [37], younger age, treatment motivation and expectations, external barriers to treatment access (e.g. work engagements [38]), personality functioning [39], comorbid PD traits [40], and mentalizing capacity [41]), but reliable, meaningful, or consistently reproducible moderators remained unidentified. This is not surprising, as most efforts have focused on single or multiple variables each in isolation [42]. Furthermore, several studies have demonstrated that incorporating multiple patient characteristics into a model simultaneously yields more accurate and robust predictions of differential treatment response than relying on a single variable [36, 43]. One promising specific statistical method enabling personalised treatment selection is the Personalised Advantage Index (PAI), introduced by DeRubeis et al. [44]. This algorithm identifies and combines multiple treatment moderators to generate individualised treatment recommendations.

The utility of the PAI in matching patients to their optimal treatment has been supported by several secondary analyses of RCTs [36]. For example, Keefe et al. [45], in a reanalysis of the McMain et al. [29] RCT comparing DBT and GPM for individuals with BPD, found that patients with high psychiatric symptom severity and impulsivity derived greater benefit from GPM. All the while, those reporting higher levels of dependency, greater psychosocial impairment, and a history of childhood emotional abuse benefited more from DBT. The five identified predictors each independently interacted with treatment, significantly predicting treatment outcome and remaining significant when combined in one omnibus model. Overall, Keefe et al. [45] found that participants who received their PAI-predicted optimal treatment experienced significantly better outcomes than those who received their non-optimal treatment, especially when the prediction was relatively stronger with a medium-to-large effect size. Meanwhile, no *single* variable was a large-effect predictor of differential treatment response. These findings suggest that identifying and combining patient characteristics that predict differential treatment response could support a more personalised treatment allocation and improvement of treatment response, consequently facilitating the optimal use of limited specialist resources.

Another important consideration is the continued reliance on traditional categorical models of PDs of almost all studies, despite the introduction of dimensional approaches in Section III of the Diagnostic and Statistical Manual of Mental Disorders (5th ed.) [46], and more recently, the adoption of a dimensional conceptualisation of PDs in the International Classification of Diseases (11th ed.) [47]. The core shift from categorical to dimensional models is a transition from a typological framework (e.g. borderline PD, antisocial PD) to one that primarily distinguishes personality disorders based on severity levels (moderate, severe, extreme PD). As the evidence supporting the clinical utility of the AMPD solidifies [48–51], there is an urgent need for studies that evaluate treatment outcomes across subgroups defined by severity of personality functioning, and studies that assess treatment efficacy using dimensional constructs of personality pathology [52, 53].

The main objective of the present study (P-DAET) is to demonstrate the non-inferiority of a generalist treatment (GIT-PD) compared to two widely implemented specialist treatments in regular clinical practice in the Netherlands—MBT and ST—in patients with severe personality disorder, as defined by the dimensional framework of the Alternative Model for Personality Disorders (AMPD). Additionally, the study will apply the PAI to explore optimal treatment allocation across both generalist and specialist treatment conditions and address direct and long-term cost differences between conditions. By combining a dimensional diagnostic framework with a personalised treatment allocation approach, this study aims to contribute to more optimally allocated, effective, and scalable interventions for individuals with severe personality disorders, ultimately supporting better clinical outcomes and more efficient use of scarce mental health resources.

### Explanation for choice of comparator {9b}

Specialist psychotherapies are currently recommended in national and international guidelines as first-choice treatment for adults with severe PD, more specifically referring to DBT, MBT, ST, and TFP [9]. Given their availability in the participating institutions, MBT and ST are included in the comparator condition. Importantly, the aim of this study lies not in comparing specific methods (e.g. GIT-PD versus MBT or GIT-PD versus ST), but in comparing generalist versus specialist approaches (e.g. GIT-PD versus MBT and ST) that adhere to predefined required conditions of implementation. These are outlined under Intervention description {15a}.

### Objectives {10}

#### Primary objective

The primary objective is to determine whether generalist treatment (GIT-PD) is non-inferior (*d* ≥ − 0.25) to specialist treatment (MBT/ST) in improving personality functioning in participants with severe personality disorders from baseline to the end of treatment (18 months; T4). In setting the non-inferiority margin, scarce evidence is available on the difference between the selected specialist treatments (MBT/ST) and no treatment [14]. However, Cristea et al. [10] demonstrated that psychotherapy is between *d =* 0.25 and *d =* 0.56 more effective than treatment-as-usual in improving BPD-relevant outcomes. Therefore, taking the statistically conservative route, the non-inferiority margin is set at *d =* − 0.25, implying that non-inferiority will be concluded if the lower bound of the confidence interval for the effect of generalist treatment relative to specialist treatment in improving personality functioning at T4 is greater than − 0.25 (Cohen’s *d*).

#### Secondary objectives

The secondary objectives are, first, to assess whether generalist treatment (GIT-PD) is non-inferior (*d* ≥ − 0.25) to specialist treatment (MBT/ST) in improving general symptom severity and psychosocial functioning in participants with severe personality disorders from baseline to the end of treatment (18 months; T4). Second, to evaluate whether generalist treatment (GIT-PD) is non-inferior to specialist treatment (MBT/ST) in participants with severe personality disorders in maintaining improvements observed at 18 months in personality functioning, general symptom severity, and psychosocial functioning from baseline to 12-month follow-up (30 months; T6). Third, to determine whether generalist treatment (GIT-PD) is more cost-effective than specialist treatment (MBT/ST) in terms of improvements in personality functioning for participants with severe personality disorders. Fourth, to explore the extent to which an algorithmically optimised combination of demographic, clinical, and psychosocial variables can predict differential treatment response (as measured by change in level of personality functioning (LPF) between baseline and end of treatment) between generalist (GIT-PD) and specialist (MBT/ST) treatments, with the expectation that combining predictors in one algorithm will outperform chance-level prediction.

## Methods: patient and public involvement, trial design

### Patient and public involvement {11}

During the design phase of the trial, the plans were presented to the study sponsor’s service user advisory board, which led to an increase in the monetary reimbursement and less extensive questionnaire packages at follow-up measurement time points for participants. Likewise, progress will be reported yearly to the service user advisory boards of the participating institutions to enable client involvement regarding trial conduct. Furthermore, plain language summaries of findings are shared with the service user advisory boards, the Dutch Centre of Expertise on Personality Disorders (Kenniscentrum Persoonlijkheidsstoornissen), and relevant mental health service user organisations, as well as published on the trial website [54] and social media.

### Trial design {12}

The P-DAET study is a pragmatic, multicentre, randomised controlled, non-inferiority trial with two parallel groups with equal group sizes (planned total *N* = 358). PD patients are randomly assigned to either generalist (GIT-PD) or specialist treatment (MBT or ST) through variable block randomisation, stratified on the mental health institution level, with a 1:1 allocation.

## Methods: participants, interventions, and outcomes

### Trial setting {13}

This multicentre trial will recruit 358 participants across 11 outpatient sites affiliated with five major Dutch mental healthcare institutions, located in both urban and rural areas across four provinces in the Netherlands: Altrecht (Zeist; Nieuwegein; Houten), Arkin NPI (Amsterdam; Amersfoort), Dimence Groep (Almelo), GGZ Oost Brabant (Helmond; Oss; Boxmeer), and de Viersprong (West-Brabant; ‘s-Hertogenbosch). New study sites in these institutes may be added to improve inclusion rates, if they provide both generalist and specialist treatments in accordance with the criteria specified under Intervention description {15a}.

### Eligibility criteria for participants {14a}

Subjects are deemed eligible if they (a) meet the DSM-5 criteria for ‘severe’ or ‘extreme’ PD according to the AMPD, as assessed by the Semi-Structured Interview for Personality Functioning – DSM 5 (STiP-5.1). A ‘severe’ PD will be diagnosed if at least two of the four facets of the STiP-5.1 are scored as ‘severe’. This mimics the proposed DSM-5 diagnostic criteria for classifying any PD, in which moderate or greater impairments in at least two out of four facets must be manifested [46]. Additionally, participants (b) need to be at least 18 years old. Exclusion criteria are (a) a primary diagnosis of autism spectrum disorder or (b) any other mental disorder diagnosed that requires prior treatment, (c) an IQ lower than 75 and/or (d) legal incapacity, as appraised during intake at the participating sites.

### Eligibility criteria for intervention deliverers {14b}

Participating sites are eligible if they offer GIT-PD and offer at least one of the specialist treatments included in this trial (MBT or ST). Treatments and therapists at participating institutions must adhere to the specified criteria described under Intervention description {15a}.

### Intervention and comparator

#### Intervention description {15a}

The experimental and comparator interventions are 1) generalist treatment (GIT-PD) and 2) specialist treatment (ST and MBT) for severe PD, respectively. Both intervention types are distinguished based upon a) level of speciality, b) format and content of the program, c) team discipline mix, d) required level of training and supervision, and e) dosage and duration of treatment.

##### Generalist condition (GIT-PD)

1. Level of Speciality: GIT-PD is a non-theoretical and non-methodological approach without a specific theory of aetiology for PD. Rather, GIT-PD builds upon treatment as usual and improves regular care by implementing a framework of common factors derived from evidence-based treatments for PD [31]. These common factors include basic skills to build a relationship (empathy, positive regard, authenticity), to address ruptures, to reach consensus on goals and tasks, and to increase (affective) self-reflection and expression of emotions, inviting the application of a wide array of therapeutic techniques with varying aetiological backgrounds (e.g. behavioural, cognitive, psychodynamic) directed at goals of the individual patient. GIT-PD focuses on 1) an engaging, validating, curious and authentic/transparent therapist stance; 2) common factors like a) therapist relationship and repair of ruptures in the alliance; b) motivation and expectancy for change; c) emotion regulation; d) self-reflection; 3) improving team functioning by targeting potentially harmful team processes and by increasing an open and transparent discussion and collaboration; 4) institutional embedment including crisis pathways and close collaboration between different services; explicit managerial support is required as a context of peer-supervision and frequent mutual support between therapists is essential for its success. GIT-PD is manualised and has been implemented across more than 30 institutions, given its simple and straightforward approach to treatment.
2. Format and Content of GIT-PD: GIT-PD consists of a structured pathway of episodes in treatment (assessment, middle, termination, booster) based upon a review of goals. GIT-PD assumes that, irrespective of PD type, all patients with PD share impairments in personality functioning as reflected in self and interpersonal impairments. GIT-PD is modularly designed. The structure of the GIT-PD programs includes: 1) an assessment phase including careful assessment, psycho-education, and discussion of diagnosis and case formulation; 2) a modular treatment phase, including group modules targeting core areas of PD, such as emotion regulation and interpersonal functioning; 3) additional personalised treatment modules that may include crisis planning, case management, trauma modules, family sessions and other problem-focused modules; 4) a follow-up phase with limited treatment focused at generalizing treatment benefits. Total treatment duration depends on specific patient needs but will usually vary between 12 and 18 months. GIT-PD may vary in format and content, allowing local variations to a certain extent. However, all treatments must be manualised, address specific problem areas of personality pathology, and aim at generalising treatment outcomes in daily life during the follow-up phase in order to prevent relapse and optimise participation in society again.
3. Team discipline mix: GIT-PD is psychotherapeutically-informed. In daily practice, treatment is supervised by licensed psychotherapists, healthcare psychologists, clinical psychologists or psychiatrists and mainly delivered by other disciplines, like psychiatric nurses, social workers and Master’s level psychologists. Training is accessible to a broad range of disciplines, too.
4. Required level of training and supervision of individual therapists: GIT-PD requires a two-day team training. Team supervision within teams will be organised according to the GIT-PD guidelines.
5. Dosage and duration of treatment: GIT-PD has a flexible approach, enabling tailoring treatment to the clinical needs of different types of patients. The treatment duration (start-end) is 12- 18 months on average.

Thus, the generalist treatment condition reflects conditions of implementation that are easier to apply in terms of required level of training, speciality, and team discipline mix, and, additionally, it reflects a more affordable treatment format in terms of content and dosage/duration.

In comparison, the specialist treatment condition reflects conditions of implementation that are required by evidence-based specialist psychotherapy, reflecting managerial conditions, required levels of training and supervision and treatment programs that resemble the investigated evidence-based programs that are recommended by guidelines.

##### Specialist condition (MBT/ST)

For the present study, we will include currently running treatment programs of MBT and ST, given their availability within the participating institutions and strong evidence base. Specialist programs must meet the following characteristics to be eligible as part of the ‘specialist, evidence-based psychotherapy’ condition:

6. Level of speciality: MBT and ST are fully manualised programs, based upon a specific theory of aetiology and pathogenesis, and both include a specific theoretical jargon and range of interventions. Both have been supported by several randomised trials and are included in all guidelines. MBT is rooted in psychodynamic and attachment theory and aims to alter personality pathology by improving mentalizing [55]. ST is an integrative treatment method, originally rooted in cognitive theory, but with additional influences from Gestalt, psychodynamic, and experiential methods [56].
7. Format and Content of Specialist treatments: We chose to include only MBT and ST programs that meet the formats of the programs that have been empirically supported through randomised trials. MBT is delivered in outpatient programs as well as day hospital programs. ST has been investigated typically in individual and group formats.
8. Team discipline mix: MBT and ST are psychotherapeutic programs. In daily practice, treatment is mainly delivered by licensed psychotherapists, healthcare psychologists, clinical psychologists and psychiatrists, although other disciplines could be part of the multidisciplinary team.
9. Required level of training and supervision of individual therapists: The licensed psychotherapists, health care psychologists, clinical psychologists and psychiatrists in the specialist programs are required to be trained in the respective therapeutic method and registered as at least, basic-level MBT therapist or junior schema therapist or in the process of acquiring this certification through supervision by an acknowledged supervisor. The basic-level MBT certification requires a 3-day training, completing between two to four MBT treatments with people with PD, and following at least 16 supervision sessions with a licensed MBT supervisor. A junior ST therapist must complete 25 hours of ST courses, 1 year of work experience with ST, at least four ST treatments consisting of at least 100 ST sessions in total, and receive at least 20 hours of supervision by a licensed ST supervisor during these treatments. On top of that, each team should include at least one certified advanced-level specialist therapist or someone in the process of obtaining certification, referring to the MBT practitioner or senior schema therapist status. To obtain MBT practitioner status, it is required that the therapist has followed the 2-day practitioner certificate training, delivered two individual and one group MBT treatment, received 20 hours of supervision by a licensed supervisor, and attained satisfactory assessment of competencies by an independent licensed supervisor. The senior schema therapist should have completed a 25-hour advanced training, 3 years of working experience, eight ST treatments, 200 ST sessions and 40 supervision sessions with a licensed supervisor. Again, individual therapists differ across the treatment sites.
10. Dosage and duration of treatment: The specialist treatment programs need to consist of a minimum of 40 sessions or a duration of 12 months, which is in line with dosage and duration of treatments evaluated in prior RCTs focusing on a population comparable to severe PD, e.g. BPD [12]. No restriction beforehand will be made on session frequency. If more than one specialist treatment (MBT or ST) or program format (e.g. intensive outpatient or day hospital MBT) is available at a site, final treatment assignment will follow the standard shared decision-making process at that site.

#### Criteria for discontinuing or modifying allocated interventions {15b}

The sponsor will suspend the study if there is sufficient ground that continuation of the study will jeopardise participant health or safety. Suspension is deemed unlikely as this study only includes regular treatment programs that have already been implemented and sustained at these sites in the Dutch mental health care system. Therapeutic progress is evaluated throughout the allocated treatment. If the therapist deems it necessary to modify the treatment, push the participant out of the allocated treatment or cross the participant over to the alternative treatment arm, then this decision needs to be discussed with the therapist in the Trial Steering Committee. Furthermore, participants can decide to discontinue the allocated treatment if desired.

#### Strategies to improve adherence to interventions {15c}

Adherence to the previously defined conditions will be measured for each site using the following criteria. For the generalist condition, the program must: a) be developed using the GIT-PD manual, b) include modules that address problem areas specific to PD, not prescribing or excluding specific techniques from other therapeutic ideologies. Instead, the GIT-PD framework implicitly and explicitly encourages therapists to use interventions that allow them to remain true to themselves by simplifying the treatment through a focus on basic principles. Furthermore, c) the integration of complete treatment programs that have a specific aetiology of PDs and are empirically supported for PDs by previous randomised studies, like MBT, ST, TFP and DBT, are excluded from the generalist condition. Additionally, the format of the generalist program should not exceed the specialist program on the same research site in terms of projected and actual treatment duration in months and dosage in minutes of treatment (per week). The projected program duration of the generalist intervention can ultimately not surpass 18 months. All the while, generalist therapists should have followed the basic GIT-PD training (2 days). Similarly, programs in the comparator/specialist condition must be designed using the treatment manual of an empirically supported MBT or ST format, including the estimated dosage and duration of the program. In addition, specialist therapists should hold a basic- or junior-level registration in their respective specialist format (MBT/ST) or be in the process of acquiring one, while at least one team member should be a fully qualified and registered MBT or advanced ST therapist, depending on the program.

For each program/team participating in the study, the following information will be collected on the organisational level: team size in staff and full-time equivalent (FTE), team composition in terms of discipline, academic level, years of experience in working with PD and MBT/ST/GIT-PD, and therapy registrations, including those for MBT and ST. For each participant, the actual duration of treatment in days, minutes of treatment, and the number of therapy sessions are obtained from the electronic patient file upon treatment completion or dropout. Furthermore, to assess the general type of institutional context and support, we will use the program adherence scale of MBT as a blueprint for identifying optimal institutional conditions for *both* specialist treatments [57], as there is no ST-specific counterpart. Additionally, we will use the quality criteria for GIT-PD from the Dutch Centre of Expertise on Personality Disorders.

#### Relevant concomitant care permitted or prohibited during the trial {15d}

Co-interventions are not offered as part of the study. Participants are, however, not explicitly prohibited from seeking additional care if desired. However, Dutch healthcare insurances cover only one PD treatment at a time. Participants are prohibited from receiving the interventions of the condition that was not allocated during randomisation for the duration of the trial.

### Outcomes {16}

The first primary outcome is the mean difference in change from baseline to end of treatment (T4) in clinician-rated personality functioning between the generalist (GIT-PD) and specialist treatment (MBT/ST) conditions, as assessed with the clinical STiP-5.1 interview. Furthermore, the second primary outcome refers to the mean difference in change from baseline to end of treatment (T4) in self-reported personality functioning between the generalist (GIT-PD) and specialist treatment (MBT/ST) conditions, based on the Level of Personality Functioning Scale – Brief Form 2.0 (LPFS-BF 2.0) questionnaire. Inclusion of the STiP-5.1 interview measure allows for blinded, clinician-rated outcome assessment, which reduces potential bias [58]. Moreover, interview measures capture a larger proportion of the variance of a latent general PD severity than self-reported questionnaires [59]. However, the use of the STiP-5.1 in psychotherapy research is novel, leading to little knowledge on the measure’s sensitivity to change [53]. Concurrently, the self-reported LPFS-BF 2.0 questionnaire has demonstrated high sensitivity to change, while its use is more established in international research [60, 61]. Therefore, the STiP-5.1 interview is given priority regarding the primary outcome, but supplemented with the self-reported LPFS-BF 2.0 questionnaire.

The secondary outcomes, among others, include the mean differences in change from baseline to the end of treatment (T4) in symptom severity (SIPP-SF, SCID-5-PQ, BSI, C-SSRS; for an outline of the here-abbreviated outcome measures, see *Plans for assessment and collection of trial data {25a})* and psychosocial functioning (WHODAS 2.0, EQ-5D-5 L, Q-LES-Q-SF) between the generalist (GIT-PD) and specialist treatment (MBT/ST) conditions. Additional secondary outcomes assess the mean differences in change from baseline to follow-up (T6) between the generalist (GIT-PD) and specialist treatment (MBT/ST) conditions in personality functioning (STiP-5.1, LPFS-BF 2.0), symptom severity (SIPP-SF, SCID-5-PQ, BSI, C-SSRS), and psychosocial functioning (WHODAS 2.0, EQ-5D-5 L, Q-LES-Q-SF). For both the short- and long-term outcomes, all available measurement points are included in the model. The secondary outcome surrounding cost-effectiveness and cost-utility of generalist (GIT-PD) versus specialist treatment (MBT/ST) addresses the incremental effects set off against the incremental costs from baseline to end of treatment (T4), calculating an incremental cost-effectiveness ratio (ICER). For the cost-effectiveness analysis, improvement in personality functioning (STiP-5.1, LPFS-BF 2.0) is set off against the incremental costs, referring to direct and indirect costs, such as treatment costs (electronic patient file data), further healthcare consumption and productivity loss (TiC-P). All the while, cost-utility is measured in quality-adjusted life years (QALYs) gained throughout the assigned intervention, calculated from EQ-5D-5L scores. The final secondary outcome measures the combined predictive value of end-of-treatment differential treatment response, based on several pre-randomisation variables. The predictive value is expressed in a personal advantage score in terms of LPF improvement from baseline (STiP-5.1, LPFS-BF 2.0). The pre-randomisation variables with prescriptive and/or prognostic value are selected and used for predicting advantage scores through the development of a PAI algorithm. For a detailed description of the instruments and the timeline of assessment, see *Plans for assessment and collection of outcomes {25a}* and *Participant timeline {18}*.

### Harms {17}

As participants follow regular treatments offered within the Dutch mental healthcare system, no increased risk of harm surrounding participation is anticipated. Unanticipated adverse events (AE) and serious adverse events (SAE) are, however, addressed in multiple ways. AEs are defined as any undesirable experience occurring to a participant during the study, regardless of whether it is considered related to the experimental or comparator interventions. An SAE is defined as any untoward medical occurrence or effect that: a) results in death; b) is life threatening (at the time of the event); c) requires hospitalisation or prolongation of existing inpatients’ hospitalisation; d) results in persistent or significant disability or incapacity; e) is a congenital anomaly or birth defect; or f) any other important medical event that did not result in any of the outcomes listed above due to medical or surgical intervention but could have been, based upon appropriate judgement by the investigator. SAEs and spontaneously reported AEs by the participant will be reported to the PI within 24 hours by the corresponding participating institution. All AEs and SAEs are recorded by the PI. SAEs must be reported to the accredited medical ethical research commission (METC Brabant) in a yearly listing.

To further increase participant safety, results on the suicidality screener (see *Columbia – Suicide Severity Rating Scale Screener/Recent Self-report (C-SSRS)*) are routinely monitored by the junior researchers. In case of a participant reporting heightened levels of suicidality, the participant’s principal therapist will be informed to prevent an SAE.

### Participant timeline {18}

Table 1 depicts the participation timeline, detailing the time points of instruments’ administration. In a pre-treatment assessment (T0p), primary and secondary outcome measures are readministered at the start of treatment in order to minimise variance in time between pre- and post-treatment measurements and account for the effect of extensive waiting list duration. Questionnaires are readministered if, due to the waiting list, ten weeks or more pass after baseline (T0b). However, the STiP-5.1 interview is readministered if 6 months have expired after baseline before the start of treatment. Participants are followed up at 3 (T1), 6 (T2), 12 (T3), 18 (T4; post-treatment), 24 (T5) and 30 months (T6).

**Table 1:**
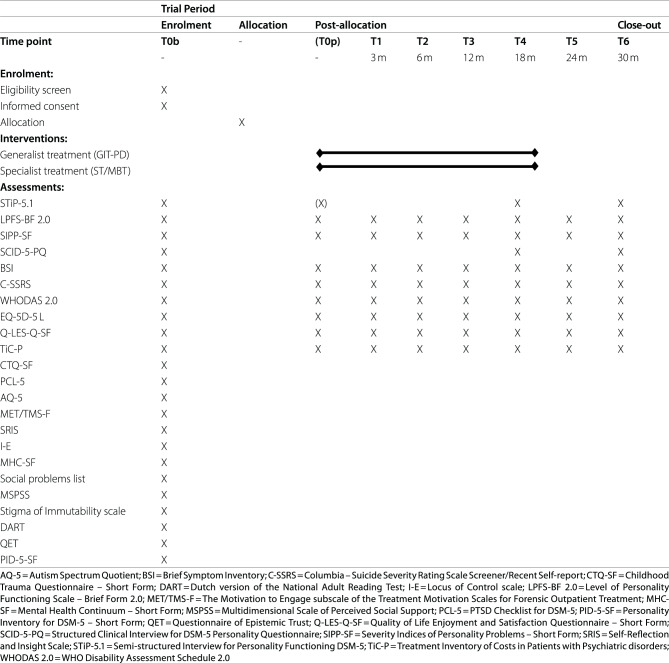
Participant timeline for the Standard Protocol Items; Recommendations for Intervention Trials (SPIRIT) {18}

### Sample size {19}

The required sample size was determined using a computer simulation. Twenty-one thousand datasets with varying sample sizes were simulated for an RCT design with seven repeated measurements on a continuous outcome variable, where both interventions exhibit equal improvement over time. The outcome measures were assumed to follow a multivariate normal distribution in both groups, with a repeated measures correlation of 0.50 and a decrease of *d* = 0.30 in both groups across the seven repeated measurements. Each of these datasets was analysed using a longitudinal multilevel model (see *Statistical methods for primary and secondary outcomes, including harms {27a}).* Subsequently, in each dataset, it was tested whether generalist treatment is non-inferior to specialist treatment by examining whether the lower limit of the two-sided 95% confidence interval of the difference between treatments in improvement over time is greater than the non-inferiority limit of *d* = 0.25. It was found that with this design, 316 participants are necessary (158 per group) to test non-inferiority with a power of 0.80 and α = 0.025. To account for attrition, which we estimate at 11.7% for the primary endpoint (T4) based on a similar study [19], the complete sample needs to consist of (316/0.883 = 357.87) 358 participants. This comes down to 179 participants per treatment condition.

### Recruitment {20}

Within each institution, all patients assessed for intake during the recruitment period appearing eligible for study participation are asked permission by the clinician to be contacted by the research team. Research staff contacts and informs the client to further discuss participation in the trial and screen for eligibility. Strategies per site vary to the extent that at Arkin NPI, the LPF, according to the STiP-5.1, is determined after intake by research staff, while all other sites implemented the STiP-5.1 into the primary intake procedure, where it is administered by trained clinicians. Treatment sites are expected to recruit varying numbers of eligible participants based on recruitment start dates, annual intake numbers, team size, and intervention capacity. Recruitment is projected to be completed by the end of 2026. A monthly inclusion forecast per site was established at the start of recruitment and will be monitored continuously. If recruitment rates fall short, corrective actions will be implemented based on site-specific factors.

## Methods: assignment of interventions

### Sequence generation

#### Roles and tools of sequence generation {21a}

Ensured concealed sequence generation and randomisation are performed through the fully automated web-based electronic data capture system of CASTOR EDC by the two unblinded junior researchers.

#### Method of sequence generation {21b}

Participants will be randomised to either the experimental or comparator condition with a 1:1 allocation. Block randomisation, with varying block sizes of 4, 6, and 8 subjects, is stratified per participating mental healthcare institution.

### Allocation concealment mechanism {22}

The block size and randomisation sequence are randomly and automatically selected. This is an ensured concealed process, as CASTOR EDC does not release the generated randomisation code. On top of that, randomisation can only be performed by the junior researchers and the outcome remains concealed to all blinded research assistants, ensuring allocation concealment.

### Implementation {23}

Participants who meet the inclusion criteria and give informed consent are added to CASTOR EDC by the research assistants. Here, participants have their own file where all outcomes are logged. All participants are randomised in CASTOR EDC after completion of the baseline measurements. CASTOR EDC does not release the generated randomisation code, ensuring that research assistants involved in enrolment and junior researchers responsible for executing treatment allocation are blind to the future order of assignments.

### Blinding

#### Who will be blinded {24a}

Treatment providers and participants cannot be blinded due to the nature of the interventions, e.g. containing psychoeducation directly related to and explained within the framework of the intervention, leading to automatic unblinding of participants. Understandably, therapists are unequivocally aware of the intervention type they are applying. Appointments for the STiP-5.1 outcome assessments will be scheduled by unblinded research staff members for blinded research staff. Participants are asked not to disclose their allocation during the appointment. Thus, staff members administering the interview at post-treatment and follow-up timepoints will be blinded. This is further ensured through CASTOR EDC roles and permissions functions.

When data is exported from CASTOR EDC, an unblinded data manager will pseudonymise the conditions. Afterwards, the data is processed, analysed, and interpreted by blinded data managers, statisticians, junior researchers, and conclusion makers. The pseudonymisation of the data will only be undone after conclusions are drafted. In this way, statisticians and conclusion makers are ensured to be blinded. The junior researchers are not blinded during data collection to enable effective day-to-day management of the trial.

#### Achievement of similarity of interventions for blinding {24b}

For post-randomisation interviews, participants are asked not to disclose their received intervention to the outcome assessor. After the interview appointment, the outcome assessor completes a set of questions to assess the extent to which blinding may have been compromised. No further procedures are in place to enhance similarity between interventions to facilitate blinding.

#### Procedure for unblinding if needed {24c}

N/A. There are no circumstances under which it is necessary to unblind the above-mentioned research staff.

## Methods: data collection, management, and analysis

### Data collection methods

#### Plans for assessment and collection of trial data {25a}

By default, self-report questionnaires are administered digitally and largely automatically through CASTOR EDC. Still, completing questionnaires in a research location and/or using pen-and-paper forms remains possible, if preferred. If participants opt for pen-and-paper forms, data is transferred manually by a research assistant into CASTOR EDC. The original forms are scanned, stored locally and compared to the transferred data in CASTOR EDC by a second research assistant to prevent erroneous answer logging. The STiP-5.1 and the Dutch Reading Test for Adults (DART) are administered by trained assessors either in person or through video call. Participant scores are logged during or directly after assessment in CASTOR EDC by the assessor. If, due to the waiting list, ten weeks or more pass between baseline measurement and the start of treatment, part of the primary instruments is readministered in a pre-treatment measurement (see Table 1). Likewise, the STiP-5.1 is readministered if 6 months have expired before the start of treatment. The original versions of all instruments used are either subject to copyright of the original authors or can be shared upon request.

#### Primary outcome measures

##### Semi-structured Interview for Personality Functioning DSM-5 (STiP-5.1)

The Semi-structured Interview for Personality Functioning DSM-5 (STiP-5.1) measures criterion A of the alternative model for personality disorders (AMPD), which pertains to the LPF consisting of self and interpersonal functioning [62]. Both self and interpersonal functioning domains can be divided into two facets and six subfacets. For self-functioning, a) Identity (i. uniqueness, ii. self-esteem, iii. emotions) and b) Self-direction (iv. goals, v. norms, vi. self-reflection) can be distinguished. Likewise, interpersonal functioning consists of c) Empathy (vii. understanding others, viii. perspectives, ix. impact) and d) Intimacy (x. connection, xi. closeness, xii. mutuality). Clinical impression of the respondent’s answers on the subfacet level enables interviewers to allot functioning scores ranging from 0 (Little to no impairment) to 4 (Extreme impairment) and to estimate aggregate scores on the facet, domain and total level, also ranging from 0 to 4. It takes 45 minutes on average to administer the interview. A total score between 0 and 48 is given to indicate the participant’s global level of personality functioning. The instrument’s internal consistency for the domain scores and total score is high (Self α = 0.94; Interpersonal α = 0.94; total α = 0.97) [62]. Furthermore, satisfactory interrater reliability and construct validity were found. However, no research has been conducted on the sensitivity to change of the measure. In the current study, assessors will be trained through (online) courses, booster classes, and supervision. Interrater reliability will be calculated for part of the readmissions, using a random selection of baseline-pretreatment assessment pairs.

##### Level of Personality Functioning Scale – Brief Form 2.0 (LPFS-BF 2.0)

Similar to the STiP-5.1, the Level of Personality Functioning Scale – Brief Form 2.0 (LPFS-BF 2.0) measures criterion A of the AMPD. Each item reflects a basic underlying impairment related to the 12 features of personality functioning according to Criterion A of the AMPD, such as identity, self-direction, intimacy, and relational functioning, resulting in a total questionnaire length of 12 items [60]. The LPFS-BF 2.0 offers a severity index for personality pathology, making it suitable for measuring treatment effects. Each item is scored on a scale from 1 (= “Very false or often false”) to 4 (= “Very true or often true”). Therefore, participants achieve a total score between 12 and 48, representing the severity of impairment in personality functioning. Weekers et al. [60] found that the scale possessed acceptable internal consistency (total scale α = 0.82; self-functioning subscale α = 0.79; interpersonal functioning subscale α = 0.71) and construct validity, as well as high sensitivity to change. Furthermore, this version of the LPFS is recommended by the International Consortium for Health Outcomes Measurement (ICHOM) as an outcome in personality disorder research [61].

#### Secondary outcome measures

##### Severity Indices of Personality Problems – Short Form (SIPP-SF)

The Severity Indices of Personality Problems – Short Form (SIPP-SF) is a dimensional self-report questionnaire measuring the severity of personality pathology by assessing the core components of adaptive and maladaptive personality functioning. This is captured in the five facets of self-control, identity integration, responsibility, relation capacities, and social concordance [63]. The questionnaire was designed in line with the concept of personality disorders as defined in the Alternative Model for Personality Disorders. In answering the 60 items, a 4-point Likert scale is used that ranges from 1 ( = fully disagree) to 4 ( = fully agree). The SIPP-SF has been shown to be sensitive to changes, in particular the facets of self-control, identity integration, and relational capacities. Furthermore, the instrument has excellent internal consistency (α = 0.75–0.91) and satisfactory validity [64].

##### Structured Clinical Interview for DSM-5 Personality Questionnaire (SCID-5-PQ)

The SCID-5-PQ is the self-report screener that belongs to the Structured Clinical Interview for DSM-5 Personality Disorders (SCID-5-PD) [65]. The questionnaire uses 106 yes/no items to screen all symptoms of the categorical personality disorder diagnoses. The screener tends to elicit many false positives and few false negatives, making it unsuitable as a stand-alone instrument in classifying personality disorders. Hence, in the current study, the questionnaire is solely used to track subjective symptom change as operationalised in Section II of the DSM-5-TR [66].

##### Brief Symptom Inventory (BSI)

The Brief Symptom Inventory (BSI) consists of 53 items, which are answered using a Likert scale comprising five levels (0–4; “Not at all” to “Extremely”). This questionnaire was developed for clinical settings and measures distress and typical psychopathological symptomatology, including somatisation, obsessive-compulsivity, interpersonal sensitivity, depression, anxiety, hostility, phobic anxiety, paranoid ideation, and psychoticism [67]. A global severity index and subscores on all 9 domains are calculated by summing the values on all items and dividing by the number of items. De Beurs & Zitman [68] showed the scale has excellent internal consistency for the Global Severity Index (GSI; α = 0.96). The subscales’ internal consistency ranges from acceptable to good (α = 0.71–0.87), while having a modest sum of items per scale. Test-retest reliability over a short period is promising (GSI *r* = 0.90, subscales *r* = 0.71–0.89). An adequate sensitivity for measuring therapeutic change has been recorded simultaneously. Moreover, the BSI has satisfactory convergent and divergent validity.

##### Columbia – Suicide Severity Rating Scale Screener/Recent Self-Report (C-SSRS)

The Columbia – Suicide Severity Rating Scale Screener/Recent Self-report (C-SSRS), developed by Posner et al. [69], assesses suicidal ideation using six items. The instrument exhibits excellent internal consistency (ordinal α = 0.98) and satisfactory construct validity (predictive and concurrent) [70, 71]. Also, this version of the C-SSRS is recommended by ICHOM as an outcome in personality disorder research [61].

##### Who Disability Assessment Schedule (WHODAS 2.0)

The 12-item WHO Disability Assessment Schedule (WHODAS 2.0) aims to measure the level of functioning on six domains: cognition, mobility, self-care, getting along, life activities, and participation. Respondents use a 5-level Likert scale to disclose how much difficulty they experience in the above-mentioned domains, ranging from “None” to “Extreme or cannot do” [72]. Saltychev et al. [73] systematically reviewed the psychometric properties of the WHODAS 2.0 in a diversity of samples and found the internal consistency to range from *α* = 0.82 to *α* = 0.95, which can be interpreted as good to excellent. Axelsson et al. [74] demonstrated good test-retest reliability (*r* = 0.83) and good construct validity. Additionally, the WHODAS 2.0 is part of the standard tool set advised by ICHOM for research on personality disorders [61].

##### Quality of Life (EQ-5D-5 L)

The EQ-5D-5 L measures decrements in health using five dimensions (mobility, self-care, usual activities, pain/discomfort, anxiety/depression), answered through a 5-point Likert scale ranging from “no problems” to “unable to” [75]. In their review, Feng et al. [76] found satisfactory test-rest reliability at the index level. Moreover, the authors conclude that the questionnaire possessed acceptable convergent and known-groups validity.

##### Quality of Life Enjoyment and Satisfaction Questionnaire – Short Form (Q-LES-Q-SF)

The Quality of Life Enjoyment and Satisfaction Questionnaire – Short Form (Q-LES-Q-SF) covers 16 items, which are answered in a 5-point Likert scale (“not at all or never” to “frequently or all the time”), to assess quality of life in the domains of physical health, subjective feelings, leisure activities, satisfaction with medication, and overall life satisfaction [77]. The short variant was extracted from the original 93-item Q-LES-Q by Endicott et al. [78]. The Q-LES-Q-SF has excellent internal consistency (*α* = 0.90) and test-retest reliability (*r* = 0.93) [77]. Furthermore, the scale has demonstrated strong convergent and discriminant validity [79].

##### Treatment Inventory of Costs in Patients with Psychiatric Disorders (TiC-P)

The Treatment Inventory of Costs in Patients with Psychiatric disorders (TiC-P) assesses healthcare consumption and productivity loss using 57 items, enabling the economic evaluation of mental illness and healthcare [80, 81]. Bouwmans et al. [80] showed satisfactory construct validity and test-rest reliability in estimating healthcare use of different provider groups (*ICC* two-way mixed models, absolute agreement = 0.57–0.88), except for the alternative medicine dimension (ICC = 0.231).

#### Predictors

The aforementioned secondary instruments assessed at baseline will also serve as candidate predictors in the PAI analysis. These candidate predictors have been selected based on previously identified predictors in comparable analyses [45, 82], as well as alignment with variables examined in ongoing or upcoming trials on optimal treatment selection for personality disorders [83–86]. Moreover, their inclusion allows for replication and verification of related findings, enhancing the robustness of predictor research surrounding PD.

##### Demographic and Clinical Characteristics

At baseline, participants answer questions regarding their biological sex, identified gender, age, ethnicity, education level, (potential) custody of children, marital and relationship status, sexual orientation, living situation, religion, employment, net income, income source(s), and the number of prior therapies received for which complaints, and within which theoretical framework. Any DSM classifications the participant has been diagnosed with at intake by the participating institution are retrieved from their electronic patient file.

##### Childhood Trauma Questionnaire – Short Form (CTQ-SF)

The Childhood Trauma Questionnaire – Short Form (CTQ-SF) employs 25 items to assess childhood trauma and distinguish between emotional neglect, physical neglect, emotional abuse, physical abuse and sexual abuse. Items are scored on a 5-point Likert scale, ranging from 1 (“Never true”) to 5 (“Very often true”) [87]. Internal consistency is found to be acceptable in a Dutch sample (*α *= 0.63–0.95) [88] and in a sample diagnosed with personality disorders (*α* = 0.81–0.91) [89]. The scale shows good factorial and convergent validity [88, 89].

##### PTSD Checklist for DSM-5 (PCL-5)

The post-traumatic stress disorder (PTSD) Checklist for DSM-5 (PCL-5) aims to screen for post-traumatic stress disorder using 20 items that correspond to the DSM-5 criteria [90]. Likewise, the four subscales of the instrument correspond to the DSM-5 clusters B through E for PTSD. The respondent rates the extent to which they suffer from a symptom using a 5-point Likert scale, ranging from 0 (“Not at all”) to 5 (“Extremely”). The instrument possesses good to excellent internal consistency (total scale *α* = 0.96, subscales *α* = 0.83–0.91) and satisfactory convergent/divergent validity [91].

##### Autism Spectrum Quotient (AQ-5)

The Autism Spectrum Quotient (AQ-5) is a very brief (5-item) screener, which has been derived from the AQ-10 [92] as a more psychometrically sound adaptation [93]. The AQ-5 measures social communication deficits, corresponding to Criterion A of the ASD diagnosis. The scale has acceptable internal consistency (*α* = 0.75) and convergent validity.

##### The Motivation to Engage Subscale of the Treatment Motivation Scales for Forensic Outpatient Treatment (TMS-F)

The Motivation to Engage (MET) subscale consists of 16 items and originates from the Treatment Motivation Scales for Forensic Outpatient Treatment (TMS-F). The MET subscale measures the participant’s commitment and readiness necessary for treatment to be successful [94]. Drieschner and Boomsma [94, 95] found the subscale possesses good internal consistency (*α* = 0.88) and adequate predictive and convergent validity.

##### Self-Reflection and Insight Scale (SRIS)

The Self-Reflection and Insight Scale (SRIS) uses 20 items to measure both inspection and evaluation (self-reflection; 12 items) and clarity of understanding (insight; 8 items) of one’s thoughts, feelings, and behaviour. Grant et al. [96] found good internal consistency (SRIS-SR *α* = 0.91; SRIS-IN *α* = 0.87), test-retest reliability (SRIS-SR *r* = 0.77; SRIS-IN *r* = 0.78), and convergent validity.

##### Locus of Control Scale (I-E)

The Locus of Control scale (I-E) assesses whether the locus of control is more internal or external, meaning to what extent a respondent perceives they can personally influence outcomes of events. The scale covers 10 items using a 5-point Likert scale [97]. Kamphuis and Emmelkamp [98] reported questionable but acceptable internal consistency (*α* = 0.66), while Sanderman [97] demonstrated adequate construct validity.

##### Mental Health Continuum – Short Form (MHC-SF)

The Mental Health Continuum – Short Form (MHC-SF) was developed by Keyes [99] to measure (hedonic and eudaimonic) well-being, which can be seen as a separate, but related, dimension to mental “ill-being” [100, 101]. The questionnaire contains 14 items divided into three dimensions: emotional well-being (EWB; 3 items), psychological well-being (PWB; 6 items), and social well-being (SWB; 5 items). Items can be answered using a Likert scale ranging from 0 (“Never”) to 5 (“Every day”). Validation of the instrument in a psychiatric sample confirmed the three-factor structure and revealed good internal consistency (total *α* = 0.92; EWB *α* = 0.88; PWB *α* = 0.85, SWB *α* = 0.77) [100].

##### Social problems list (IAPT)

Seventeen areas of social problems, such as unemployment and social isolation, are logged using yes/no items of the Social Problems list derived from the Improving Access to Psychological Therapies (IAPT) program [102]. An 18^th^ item provides the opportunity to log additional social problems not covered by the seventeen areas previously inquired. No prior psychometric validation has been executed.

##### Multidimensional Scale of Perceived Social Support (MSPSS)

The Multidimensional Scale of Perceived Social Support (MSPSS) questionnaire measures the perceived social support from family, friends, and a significant other [103, 104]. The 12 items are answered using a 7-point Likert scale ranging from “Very strongly disagree” to “Very strongly agree”. For the Dutch translation, Pedersen et al. [105] found the questionnaire to have excellent internal consistency (*α* = 0.94) and construct validity.

##### Stigma of Immutability Scale

For their multicentre RCT, Wibbelink et al. [86] developed a scale to assess the extent to which people believe their (borderline) personality disorder is resistant to treatment. The scale uses 7-point Likert scales to answer 5 items. No prior psychometric validation has been executed. Composite scores will be computed for each factor revealed by an exploratory factor analysis applied to the 5 item scores.

##### Dutch Version of the National Adult Reading Test (DART)

The Dutch version of the National Adult Reading Test (DART; [Nederlandse Leestest voor Volwassenen]; NLV) can be used to estimate (verbal) intelligence through assessment of the pronunciation of 50 words with irregular spelling [106]. The test has high internal consistency (*α* = 0.90) [107], inter-rater (*r* = 0.98) and retest reliability (*r* = 0.98) [108], as well as good criterion validity [109].

##### Questionnaire of Epistemic Trust (QET)

By administering the 24-item Questionnaire of Epistemic Trust (QET), insight is gained into a person’s developmental capacity to accept information as authentic, trustworthy, generalizable, and relevant to the self [110]. Items are answered using a 5-point Likert scale ranging from 1 (“Totally agree”) to 5 (“Totally disagree”). The instrument sheds light on four factors referring to hypervigilance, curiosity/openness, expectation of help, and openness to help. The QET has good to excellent internal consistency (total score *α* = 0.91, subscales *α* = 0.80–0.90), while also possessing satisfactory convergent/divergent validity [110].

##### Personality Inventory for DSM-5 – Short Form (PID-5-SF)

The Personality Inventory for DSM-5 – Short Form (PID-5-SF) assesses pathological personality traits across five domains and 25 facets [111–113]. A total of 100 items is answered on a 4-point scale ranging from 0 (“Very false”) to 3 (“Very true”) [111]. The measure has satisfactory construct and criterion validity, as well as good internal consistency on both the domain level (*α* = 0.89–0.91) and the facet level (*α* = 0.74–0.88) [113].

#### Plans to Promote Participant Retention and Complete Follow-Up {25b}

Participant retention is promoted through multiple strategies aimed at a personalised approach that minimises burdens and maximises accessibility [114, 115]. Whenever possible, existing data is reused to avoid redundant assessments. For example, STiP-5.1 interviews conducted during regular intake are repurposed for eligibility assessment and baseline measurements. Routine outcome monitoring (ROM) is adapted to prevent overlap with study instruments and again supplemented with SIPP-SF and BSI results at designated time points to facilitate usage during treatment trajectories. Before each assessment, research staff call participants to discuss their availability and preferred mode of completion (online or in-person), ensuring a participant-centred approach. Online assessment is set as the default to enhance convenience. Furthermore, participants receive a total reimbursement of €50, distributed across T1 (€15), T4 (€20), and T6 (€15). If a participant considers dropping out, research staff will encourage continued participation by offering a reduced test battery focused on collecting the primary outcomes at T4 and T6. The extent of this set depends on the participant’s willingness.

### Data Management {26}

All study data is centrally managed and securely stored for the duration of the trial in CASTOR EDC and for 15 years after trial completion. All data in CASTOR EDC is pseudonymised and stored on EU servers based in the Netherlands [116]. Hosting is NEN7510 compliant. Multi-factor authentication for research staff is enforced. Indefinite long-term storage on secured servers is facilitated by Tilburg University through TiU Dataverse, which is based on the Harvard University Dataverse repository.

Regarding data entry, included participants receive a study ID generated by CASTOR EDC. In all data files and during statistical analysis, participants are identified through their study ID, which can only be linked to personal data through a linking file (see *Confidentiality {33}*). All interview scores and data from electronic patient file records are entered manually by research assistants, while questionnaire responses are automatically captured during administration to participants in CASTOR EDC. When possible, data entry is checked and validated for a random case sample of entered STiP-5.1 and DART scores. Missing data during entry of questionnaires is prevented by CASTOR EDC through enforced item-based validation. Completed questionnaire packages are locked and cannot be altered. Only the junior researchers and the data manager are permitted to export data. Only the necessary research staff have access to exported data files. Exported data is checked, transformed and made anonymous on secured internal servers of Arkin GGZ, and stored and analysed on secured internal servers of Tilburg University. Data transformation is executed following automated scripts to guarantee transparency and replicability. In addition, CASTOR EDC produces a complete audit trail of all actions performed, e.g. fields updated and data exported.

### Statistical methods

#### Statistical methods for primary and secondary outcomes including harms {27a}

*Non-inferiority.* The primary research question is answered through longitudinal multilevel modelling of the short-term difference (T4; 18 months) between generalist (GIT-PD) and specialist treatment (MBT/ST) in improvement of personality functioning, following the intention-to-treat principle. Two separate multilevel mixed-effects models are used for the two primary outcomes, meaning both the STiP-5.1 at two time points and the LPFS-BF 2.0 at five time points. The model levels refer to the repeated measurements (level 1) of each individual (level 2) nested within treatment sites (level 3). Both models will be adjusted for the respective baseline outcome value, while random intercepts and slopes are modelled to account for the heterogeneous individual change trajectories over time. Furthermore, random intercepts and slopes are modelled on the treatment site level to account for heterogeneous change trajectories across sites. No further covariates will be added. Two-tailed significance levels are set at *α* = 0.05, while effect estimates are reported with corresponding 95% two-sided confidence intervals. If the required sample size is not achieved due to problems with enrolment and inclusion, all non-inferiority analyses are converted to a one-sided confidence interval approach in order to retain sufficient statistical power. In case of convergence issues, the model’s complexity will be reduced by 1) not modelling the random slope but only the random intercept at the third level of treatment site, 2) replacing treatment site with institution with regards to the third level along with the respective random intercepts and slopes, or 3) omitting the third level in the analysis altogether if necessary. Trial-level non-inferiority will be assessed using a fixed-sequence testing procedure [117], prioritising the clinician-rated LPF (STiP-5.1) due to its blinded assessment and superior capability of measuring latent PD severity [58, 59]. If non-inferiority is demonstrated on the STiP-5.1, the self-reported LPFS-BF 2.0 will be tested subsequently to provide additional supportive evidence, given its high sensitivity to change [60]. If non-inferiority is not demonstrated on the STiP-5.1, the LPFS-BF 2.0 will not be formally tested as a primary outcome, and non-inferiority cannot be concluded at the trial level. This hierarchical approach preserves statistical power while controlling the family-wise error rate across multiple endpoints and prioritising blinded assessment to minimise potential bias.

To answer the secondary research questions regarding long-term (T6; 30 months) non-inferiority of generalist (GIT-PD) versus specialist treatment (MBT/ST) in improving personality functioning, an identical intention-to-treat longitudinal multilevel model will be fit as for the primary outcome, while including all available repeated measures. This ultimately results in the inclusion of three and seven repeated measures for the STiP-5.1 and LPFS-BF 2.0, respectively. Likewise, to assess the short-term (T4; 18 months), as well as the long-term (T6; 30 months) non-inferiority of generalist (GIT-PD) versus specialist treatment (MBT/ST) in improving symptom severity and psychosocial functioning, the same intention-to-treat longitudinal multilevel model will be applied for each of the secondary study parameters (SIPP-SF, SCID-5-PQ, BSI, C-SSRS, WHODAS 2.0, EQ-5D-5 L, Q-LES-Q-SF). Likewise, for the short-term outcome, the five repeated measures (level 1) of each individual (level 2) are nested within treatment sites (level 3). Models are adjusted for the respective outcome’s baseline value, random intercepts, and slopes are modelled at the second and third level to account for heterogeneous individual and treatment site change trajectories over time, and two-tailed significance levels are set at *α* = 0.05, while effect estimates are reported with corresponding 95% two-sided confidence intervals. The non-inferiority margin is set at *d* = − 0.25. On top of that, all AEs and SAEs are descriptively reported for both treatment arms.

*Cost-effectiveness.* To assess the cost-effectiveness of treatments, we will conduct a comprehensive economic evaluation consisting of a cost-effectiveness analysis (CEA) and a cost-utility analysis (CUA). The analyses will be conducted and reported following [118], the CHEERS 2022 statement [119] and the ISPOR good research practices task force report on cost-effectiveness analysis alongside clinical trials II [120]. To evaluate the cost-effectiveness, improvement in LPF (as measured with the LPFS-BF 2.0 and STiP-5.1) is set off against costs incurred from the societal perspective. Treatment costs within the context of the participating mental health treatment centres are measured using patient file data (e.g. treatment minutes and type of therapist). Costs outside of the participating treatment centres are assessed with the TiC-P questionnaire. The TiC-P has a recall period of 3 months, which will be linearly extrapolated to complement the full intervals between time points. All cost estimates will be based on reference pricing as defined by Hakkaart-Van Roijen et al. [121].

For the CUA, health utilities are calculated using the Dutch tariff for the EQ-5D-5 L [122]. These utility scores will be used to calculate quality-adjusted life years (QALYs). QALYs gained between baseline measurements and the primary endpoint at 18 months after the start of treatment are the outcome variable for the CUA. This outcome variable is defined as the number of life years gained adjusted for quality of life. By projecting QALYs gained per condition as an operationalisation of utility, the cost-utility of the assigned intervention will be determined. In sum, we aim to execute the CEA and CUA from the societal perspective based on intention-to-treat analyses with an estimated time-horizon of 18 months after T0 for the economic evaluation on the primary endpoint.

For both the CEA and CUA, the incremental costs (electronic patient file data and TiC-P) and the incremental effects (change in LPF as measured with the LPFS-BF-2.0 and STiP-5.1 for the CEA; QALYs as measured with the EQ-5D-5 L for the CUA) will be used to calculate incremental cost-effectiveness ratios (ICER). The uncertainty and robustness of the ICER will be analysed using bootstrapping. Next, the bootstrapped ICER will be plotted on cost-effectiveness planes where the horizontal axis displays the difference in effectiveness and the vertical axis displays the difference in costs. Further, the ICER will be used to examine willingness-to-pay (WTP) levels and then will be plotted in cost-effectiveness acceptability curves, displaying the cost-effectiveness of the experimental condition compared to the active control condition. Hence, WTP analysis will be conducted to determine the incremental costs per incremental unit of effect (QALYs or change in LPF) gained compared to WTP thresholds. A fitting discount rate will be applied for outcomes and future costs in accordance with NICE guidelines [123] and Drummond et al. [118]. Uncertainty in results will be assessed using both bootstrapping and sensitivity analyses. Variability and heterogeneity analyses will be conducted. Potential subgroups based on patient characteristics will be part of exploratory analyses.

*Personalised Advantage Index*. In examining what treatment works better for whom, an algorithm will be built that predicts differential treatment responses regarding LPF between generalist and specialist treatment. The algorithm quantifies the magnitude by which either treatment is estimated to outperform the other on the participant level, using the Personalised Advantage Index (PAI). The PAI was initially developed by DeRubeis et al. [44], but recently Meinke et al. [124] revised the algorithm, addressing key analytical issues in the traditional approach. As a result, this Advanced PAI approach introduced less risk of bias and prevented data leakage, thus providing more precise estimates while minimising the risk of overestimating model performance. Following Meinke et al.’s [124] guidance, PAI analyses will be conducted using their open-source code. This means a 5-fold cross-validation with 100 iterations will be utilised, covering not only the data preprocessing steps in terms of candidate predictor exclusion, imputation, and selection but also training, testing, and evaluating the performance of the models. More specifically, candidate predictors will be excluded if more than 30% of values are missing, while imputing missing values for the remaining candidate predictors. Herein, the mode will be used for categorical and binary variables, while multivariate imputation by chained equations (MICE) is going to be used for numerical variables. Furthermore, predictors lacking variance and binary predictors with 90% of participants or more in either category will be excluded. Multicollinearity between candidate predictors is operationalised as Pearson correlation (numerical variables) or Jaccard similarity (binary variables) of  > 0.75. Either of two similar candidate predictors should be removed by excluding the variable with the highest mean correlation/similarity with the remaining predictors. Candidate predictor exclusion will be repeated until no correlation/similarity of  > 0.75 is observed.

Subsequently, distinct prediction models are to be fitted for the generalist and specialist treatment. First, predictors will be selected using Elastic Net regression, which is a supervised machine-learning method that combines the penalised linear Lasso and Ridge regressions to identify a parsimonious and stable subset of predictors. Then, Ridge regression will be used to fit the final prediction models using the selected predictors per treatment. For the participants in the test-fold of the 5-fold cross-validation, the resulting prediction models will be used to calculate a ‘factual’ prediction for the treatment actually received and a ‘counterfactual’ prediction for the treatment not actually received. To quantify the performance of the prediction models for both treatments, the outcome of the factual prediction will be compared to the actual LPF at post-treatment in terms of the mean absolute error (MAE), the root mean square error (RMSE), and the correlation.

The PAI scores are then derived from subtracting the counterfactual prediction from the factual prediction to discern whether a participant received their optimal or non-optimal treatment. Negative and positive PAI scores refer to having received the optimal and nonoptimal treatment, respectively. The PAIs utility in guiding treatment selection will be evaluated through comparing LPF at post-treatment for participants having received their optimal versus nonoptimal treatment using an independent one-sided t-test. Additionally, Cohen’s *d* will be computed for the mean difference. As per customary practice in PAI analyses, this evaluation should be repeated for the 50% of participants with the largest absolute PAI scores to determine utility for those with the strongest predictions. The Advanced PAI approach will be performed for both the clinician-rated (STiP-5.1) and the self-reported LPF (LPFS-BF 2.0).

#### Definition of analysis population {27b}

Following the intention-to-treat principle, participants will be analysed according to the assigned intervention arm, disregarding actual compliance with the assigned intervention. Furthermore, in inspecting the robustness of the noninferiority outcomes, supportive sensitivity analyses are carried out per-protocol, meaning only participants who did not drop out of the assigned intervention are included.

#### How missing data will be handled in the analysis {27c}

The multi-level mixed-effects models allow for incomplete outcome data, while assuming Missing at Random (MAR). The actual missingness mechanism is, however, unknown. This will be examined by comparing baseline characteristics between participants with and without missing outcome values. Furthermore, to examine the influence of missingness on the primary analyses, two sensitivity analyses are performed; the first repetition of the primary analyses will additionally use multiple imputation through predictive mean matching to impute the missing values on the repeated measurements of the dependent variable. The second repetition will cover a complete case analysis, including only the participants without missing data on the respective primary outcome.

#### Methods for any additional analyses (e.g. subgroup and sensitivity analyses) {27d}

N/A. Other than the above-mentioned (sensitivity) analyses, no additional analyses are planned.

## Methods: Monitoring

### Data monitoring committee

#### Composition of data monitoring committee (DMC) {28a}

N/A. A data monitoring committee is unwarranted for the purposes of this study, as no additional risk of harm is anticipated for participation. Moreover, no periodic data monitoring is carried out, except for the recruitment and inclusion progress performed by the TSC.

#### Explanation of any interim analyses and stopping guidelines {28b}

N/A. No interim analyses are specified, as no additional risks are expected for participation in the current trial.

### Trial monitoring {29}

Trial conduct is audited in multiple ways. Firstly, both the Medical Ethical Research Commission (METC Brabant) and the funder of the study (Foundation for VCVGZ Support) will be informed about the progress of the trial on a yearly basis by the PI. Secondly, the quality of collected data in CASTOR EDC is guaranteed through its integrated audit trail and will be assessed shortly after the start of data collection by the data manager. Thirdly, multiple participating institutes (Arkin NPI, GGZ De Viersprong, GGZ Oost Brabant) are subject to an internal and external independent audit cycle, in accordance with NEN-EN 15224, to ensure the quality of their TOP Clinical Psychiatric Care, specialist diagnostic research and treatment programs for people with complex psychiatric problems and PDs. Lastly, the trial steering committee has monthly meetings to oversee the trial conduct.

## Ethics

### Research ethics approval {30}

Ethical approval for P-DAET was issued by the METC Brabant (NL86263.028.24). Moreover, a data protection impact assessment (DPIA; TSB_1632) was conducted by Tilburg University’s Ethics and Review Board. Additionally, permission was sought from the participating institutions’ advisory committees of scientific research. Finally, all participants will provide written informed consent.

### Protocol amendments {31}

Any (non)substantial amendments to the study protocol will be reported to and ratified by the METC Brabant and reported publicly on clinicaltrials.gov. Further, the METC Brabant can impose follow-up steps after amendments.

### Consent or assent

#### Who will obtain informed consent {32a}

After eligibility screening, patients are informed by research staff both verbally and in writing. After at least a week, to ensure sufficient time for consideration, an informed consent appointment is planned either at a research site or via video conference. During this appointment, the prospect’s questions are discussed and, if applicable, the consent form is signed digitally or in writing by the prospect. Consent forms that are signed on paper at home during a video call conference are sent back by participants via conventional letter mail. Once received, the researcher or their representative signs the form, after which it is stored in a decentralised manner, and a copy is provided to the participant. The model consent form in Dutch is available upon request from the corresponding author.

#### Additional consent provisions for ancillary studies {32b}

The consent form specifically seeks permission for the use of participants’ data in ancillary studies and recruitment for follow-up studies. Prospects can still participate in the current study if they decline participation in ancillary or follow-up studies.

### Confidentiality {33}

During recruitment, eligibility screening, data collection, and archival, personally identifiable data is collected within the institutions from electronic patient files and securely stored at each participating institution in a separate linking file. In the linking file, each participant is linked to a pseudonym, meaning an application number or a study ID, depending on the time point (i.e. before or after inclusion). During recruitment, application, and eligibility screening, application numbers are used to communicate about participants. Signed informed consent forms are also stored in a decentralised manner at each participating institution. Access to both the linking files and consent forms is restricted to local research staff members who require it for their work, as well as any relevant supervisory authorities. After informed consent is obtained, a study ID is generated, enabling pseudonymized central data collection in CASTOR EDC. If an application does not lead to participation, all personally identifiable data is removed from the linking file, except for the original electronic patient file number and the application number. Linking files are retained at the participating institutions for 15 years after study completion, while pseudonymized research data is archived in CASTOR EDC for 15 years and in TiU Dataverse indefinitely (see *Data Management {26}*).

### Ancillary and post-trial care {34}

The studied interventions are standard treatments within the participating Dutch mental healthcare institution. Therefore, participation does not involve additional risks, and therapeutic progress is reviewed collaboratively between the participant and therapist throughout the treatment. After completing the allocated treatment, participants can seek follow-up care if desired. However, the participant’s respective institution is not allowed to provide care of the opposite treatment arm for the duration of the trial, unless explicit permission is sought and obtained from the TSC. Dropping out of the study will not affect the treatment provided. If participants discontinue the allocated treatment, they will still be assessed and included in the analysis according to the intention-to-treat principle, unless consent is withdrawn.

## Discussion

Although generalist treatments are widely used, the P-DAET trial is the first RCT assessing the non-inferiority of generalist treatment (GIT-PD) compared to specialist treatment (ST/MBT). We expect to find that, on average, generalist treatment is non-inferior to specialist treatment in terms of improving the level of personality functioning, general symptom severity and psychosocial functioning at post-treatment (18 months), as well as maintaining improvements at follow-up (30 months). Additionally, generalist treatment is expected to be more cost-effective than specialist treatment at both post-treatment and follow-up. Lastly, it is hypothesised that a combination of individual participant characteristics can predict differential treatment response above chance level, enabling the construction of a personalised treatment selection algorithm. P-DAET is, ultimately, aiming to contribute to the organisation of accessible and optimal allocation of cost-effective evidence-based psychotherapeutic care for those suffering from severe personality disorders by addressing these gaps in our current knowledge. The core clinical relevance of this study is determining whether a well-structured generalist treatment can serve as a non-inferior alternative to specialist treatment on average, while identifying which patients in particular require specialist care.

The study has several strong and innovative points. First, our study focuses on comparing treatment frameworks, rather than comparing specific treatment methods. Specifically, this study goes beyond comparing GIT-PD with MBT or ST: It operationalises the conditions needed for implementation and delivery of treatments (i.e. generalist vs. specialist), distinguishing them by the very factors that often hinder large-scale implementation of specialist treatments, referring to the required (and often costly) disciplinary mix, intensive training demands, level of specialisation and supervision, and the typically high dosage and duration of these treatments. By contrast, generalist treatments, including GIT-PD, are proposedly to be less hindered by these factors, as they require minimal additional resources beyond existing treatments-as-usual, while harnessing the common factors across PD treatments. This positions generalist treatments as substantially easier to implement, and therefore, as a potentially more accessible commodity to enhance the general level of care for individuals with severe PDs on a broader scale, compared to specialist treatment. The central question is whether this increased scalability comes at a cost. We hypothesise that, on average, it does not. However, for certain subgroups of patients—those with specific clinical profiles—specialist treatment may still be the preferred or necessary option. As such, this study aims to inform a more effective and equitable allocation of limited specialist resources, while contributing to more accessible and good-enough care for individuals with severe PDs.

Second, our study aligns with a dimensional diagnostic framework, both in specifying the target population and in evaluating treatment outcomes. Since the introduction of the Alternative Model for Personality Disorders (AMPD) over a decade ago [46], few clinical trials have adopted this framework as an outcome measure. P-DAET is among the first to do so [53]. Given the novelty of assessing impairments in self and interpersonal functioning as primary outcomes, we chose to integrate both clinician-rated and participant-reported perspectives. Each approach has distinct advantages: repeated administration of the STiP-5.1 interview allows for blinding of outcome assessors, thereby reducing potential bias [58], while being better able to capture latent PD severity than self-report measures [59]. However, its use in psychotherapy research is novel, making it unclear to what extent the measure is sensitive to change. In contrast, the LPFS-BF 2.0 questionnaire has demonstrated sensitivity to change in previous studies and is well-established in international research. It is also easy to administer repeatedly and imposes minimal burden on participants. Given these considerations, the STiP-5.1 interview will be prioritised in our primary outcome analysis, while the LPFS-BF 2.0 will supplement and contextualise findings to enhance interpretability, especially when the sensitivity to change of the STiP-5.1 appears to be insufficient. Another major strength of the present study is its inclusion of multiple relevant outcome domains, resulting in a comprehensive and clinically meaningful set of measures. These include impairments in self and interpersonal functioning, severity of traditional personality disorder (PD) symptoms, general psychiatric symptom severity, and psychosocial functioning. The integration of both more variable outcomes—such as PD symptom severity—and more stable outcomes—such as psychosocial functioning [125, 126]—is particularly valuable for capturing the broad range of clinically relevant consequences associated with severe PDs.

Still, any future results of the P-DAET trial must be interpreted within the context of potential limitations. Recently, it was argued that non-inferiority trials must feature an inactive control condition, as only then can the effectiveness of both active conditions be implied [127]. However, ethical concerns arise when considering an inactive condition, due to the long duration of treatments. With many patients already waiting extensively to start a PD treatment, sometimes for several years, randomisation of these patients to an inactive condition is deemed unfeasible and unethical. Moreover, we think that the inclusion of an inactive control condition is not necessary in the present study. The control condition—specialist psychotherapy—has consistently been shown to outperform inactive conditions across a wide range of studies employing diverse treatment methods. A substantial body of evidence demonstrates that treatment programs characterised by key features—such as a solid theoretical and methodological foundation, manualisation, delivery by well-trained professionals, and adequate dosage—are more effective than no treatment or unstructured treatment as usual [10–14]. Given this, we consider the effectiveness of specialist treatments sufficiently established, provided that they are delivered under conditions consistent with those shown to be effective in prior research. To address the potential concern that the specialist treatments in our study might not meet such standards, we have aimed to restrict inclusion to MBT and ST programs that conform to the specifications used in their respective randomised controlled trials. This includes adherence to established parameters regarding treatment duration, dosage, team composition, training requirements, format, content, and level of specialisation.

Another potential concern is the difference in average treatment duration between conditions. Indeed, treatment dosage and duration are among the defining distinctions between the generalist and specialist approaches. In line with previous trials, specialist treatments are expected to be delivered at a significantly higher dosage than the generalist condition. To ensure comparability between conditions, assessment time points are scheduled at fixed intervals, independent of treatment type. The primary endpoint is set at 18 months after treatment initiation, aligning with the projected average treatment duration for specialist treatments. While actual treatment durations may vary at the individual level, the intensive treatment phase is expected to be completed in all programs by this 18-month mark, with only low-intensity booster sessions continuing beyond this point. Therefore, the 24-month time point has been designated as the post-booster follow-up, by which time treatment trajectories in both conditions will have concluded for nearly all participants. This alignment of measurement time points ensures comparability in evaluating (cost-)effectiveness across conditions. Furthermore, the extended follow-up assessments at 24 and 30 months allow for the examination of potential delayed or sustained effects related to differences in treatment duration.

In conclusion, P-DAET will be the first randomised controlled trial to compare the non-inferiority of a generalist treatment approach (GIT-PD) directly to specialist treatments (MBT/ST) for individuals with severe personality disorders, as defined by the AMPD. This study is expected to generate novel insights into the effectiveness and real-world applicability of both generalist and specialist interventions. It will also address which patient needs are better met by more expensive specialist interventions. In this way, it contributes to a greater accessibility for all patients with severe personality disorders as well as an empirically supported assignment of these patients to different treatment types.

## Trial status

As of September 26, 2024, the trial is actively recruiting participants. Enrolment began on October 21, 2024, and is expected to continue until December 31, 2026. Data collection is anticipated to be completed by December 2029. The current protocol version is Version 04.

## Electronic supplementary material

Below is the link to the electronic supplementary material.

Supplementary Material 1

## Data Availability

No datasets were generated or analysed during the current study.

## Abbreviations

AMPD: Alternative model for personality disorders
AQ: Autism Spectrum Quotient
BPD: Borderline personality disorder
BSI: Brief Symptom Inventory
CAT: Cognitive Analytic Therapy
CEA: Cost-effectiveness analysis
C-SSRS: Columbia – Suicide Severity Rating Scale Screener/Recent Self-report
CTQ-SF: Childhood Trauma Questionnaire – Short Form
CUA: Cost-utility analysis
DART: Dutch version of the National Adult Reading Test
DSM-5: Diagnostic and Statistical Manual of Mental Disorders, Fifth Edition
DBT: Dialectical Behavioural Therapy
DPIA: Data Protection Impact Assessment
EWB: Emotional well-being
FTE: Full-time equivalent
GCC: Good Clinical Care
GIT-PD: Guideline-Informed Treatment for Personality Disorders
GPM: Good Psychiatric Management
GSI: Global Severity Index
IAPT: Improving Access to Psychological Therapies
ICD-11: Eleventh Revision of the International Classification of Diseases
I-E: Locus of Control scale
IOP MBT: Intensive outpatient Mentalisation-Based Treatment
LPF: Level of Personality Functioning
LPFS-BF 2.0: Level of Personality Functioning Scale – Brief Form 2.0
MAE: Mean absolute error
MAR: Missing at random
MBT: Mentalisation-Based Treatment
METC: Medical Ethical Research Commission
MET/TMS-F: The Motivation to Engage subscale of the Treatment Motivation Scales for Forensic Outpatient Treatment
MHC-SF: Mental Health Continuum – Short Form
MICE: Multivariate imputation by chained equations
MSPSS: Multidimensional Scale of Perceived Social Support
NLV: Nederlandse Leestest voor Volwassenen, see *DART*
PAI: Personalised Advantage Index
PCL-5: PTSD Checklist for DSM-5
PD: Personality disorder
P-DAET: Personality Disorder Access to Effective Treatment
PI: Principal investigator
PID-5-SF: Personality Inventory for DSM-5 – Short Form
PTSD: Post-traumatic stress disorder
PWB: Psychological well-being
QALYs: Quality-adjusted life years
QET: Questionnaire of Epistemic Trust
Q-LES-Q-SF: Quality of Life Enjoyment and Satisfaction Questionnaire – Short Form
RCT: Randomised controlled trial
RMSE: Root mean square error
SAE: Serious Adverse Events
SCID-5-PQ: Structured Clinical Interview for DSM-5 Personality Questionnaire
SCM: Structured Clinical Management
SIPP-SF: Severity Indices of Personality Problems – Short Form
SRIS: Self-Reflection and Insight Scale
SRIS-IN: Self-Reflection and Insight Scale – Insight subscale
SRIS-SR: Self-Reflection and Insight Scale – Self-Reflection subscale
ST: Schema Therapy
STiP-5.1: Semi-structured Interview for Personality Functioning DSM-5
SWB: Social well-being
TAU: Treatment as usual
TFP: Transference Focused Psychotherapy
TiC-P: Treatment Inventory of Costs in Patients with Psychiatric disorders
TMS-F: Treatment Motivation Scales for Forensic Outpatient Treatment
TSC: Trial Steering Committee
WHODAS 2.0: WHO Disability Assessment Schedule 2.0
